# Severe gut mucosal injury induces profound systemic inflammation and spleen-associated lymphoid organ response

**DOI:** 10.3389/fimmu.2023.1340442

**Published:** 2024-01-08

**Authors:** Xiao Wang, Chao Du, Saravanan Subramanian, Lucas Turner, Hua Geng, Heng-Fu Bu, Xiao-Di Tan

**Affiliations:** ^1^ Pediatric Mucosal Inflammation and Regeneration Research Program, Center for Pediatric Translational Research and Education, Department of Pediatrics, College of Medicine, University of Illinois at Chicago, Chicago, IL, United States; ^2^ Department of Pediatrics, Ann and Robert H. Lurie Children’s Hospital of Chicago, Feinberg School of Medicine, Northwestern University, Chicago, IL, United States

**Keywords:** DSS-model, gut injury, systemic inflammation, spleen dysfunction, lymphoid organ

## Abstract

Clinical evidence indicates a connection between gut injuries, infections, inflammation, and an increased susceptibility to systemic inflammation. Nevertheless, the animal models designed to replicate this progression are inadequate, and the fundamental mechanisms are still largely unknown. This research explores the relationship between gut injuries and systemic inflammation using a Dextran Sulfate Sodium (DSS)-induced colonic mucosal injury mouse model. Continuous treatment of adult mice with 4% DSS drinking water yielded a remarkable mortality rate by day 7, alongside intensified gut injury and detectable peripheral inflammation. Moreover, RNAscope *in situ* hybridization with 16S rRNA probe noted bacterial penetration into deeper colon compartments of the mice following treatment with DSS for 7 days. Histological analysis revealed inflammation in the liver and lung tissues of DSS-treated mice. In addition, we found that DSS-treated mice exhibited elevation of Alanine transaminase (ALT) and Aspartate transaminase (AST) in peripheral blood and pro-inflammatory cytokine levels in the liver. Notably, the DSS-treated mice displayed a dampened metabolic profile, reduced CD45 marker expression, and an increase in apoptosis within the lymphoid organ such as spleen. These findings suggest that high-dose DSS-induced gut injury gives rise to sepsis-like systemic inflammation characterized by multiple organ injury and profound splenocyte apoptosis and dysfunction of CD45^+^ cells in the spleen, indicating the role of the spleen in the pathogenesis of gut-derived systemic inflammation. Together, the severe colonic mucosal injury model facilitates research into gut damage and associated peripheral immune responses, providing a vital framework for investigating mechanisms related to clinically relevant, gut-derived systemic inflammation.

## Introduction

The intestinal epithelium, comprised of a single layer of cells, selectively permits essential nutrients, electrolytes, and water to enter the systemic circulation. Simultaneously, it blocks potentially harmful substances, such as foreign antigens and microorganisms (1, 2). Serving as a pivotal interface between the internal and external environments (3, 4), any dysfunction in this barrier can lead to the leakage of pathogens and toxins, which may then trigger systemic inflammation and sepsis (5). This premise is supported by findings of the gut leakage marker, serum (1→3)-β-D-glucan (BG), in several animal sepsis models and patients with sepsis (6). The initial exposure to bacterial products, particularly gut bacteria-derived LPS, stimulates lamina propria immune cells through the TLR4/MyD88-dependent pathway (7, 8). Additionally, bacteria-free DNA present in the blood can amplify this LPS-induced inflammation (9). As a result, pro-inflammatory mediators are released, and if they enter circulation (10), they can cause widespread inflammation affecting multiple organ systems. Despite these findings, a complete understanding of this mechanism remains elusive, essentially because animal models do not perfectly emulate this clinical process.

The cecal ligation and puncture (CLP) model, extensively utilized by our group and others (11, 12), serves as an *in vivo* representation for studying systemic inflammation, sepsis, and multiple organ dysfunction (MOD). It simulates polymicrobial pathogenesis and produces cytokine changes analogous to those observed in humans, with symptoms emerging within 12–24 hours (13). On the other hand, Lipopolysaccharides (LPS), a non-bacterial endotoxin, is employed to induce sepsis, exhibiting symptoms within just a few hours (14). Significant advancements have been achieved with the LPS model, emphasizing the potential role of increased gut permeability in severe inflammation. A key finding from this research is the crucial role of iron recycling in the inflammation process (15). Both the LPS and CLP models are essential for understanding the interplay between systemic inflammation and multi-organ interactions (16, 17), especially concerning the intestine (14, 18, 19). While both models display elevated levels of pro-inflammatory cytokines like IL1β, TNFα, and IL6, only the LPS model reveals a protective effect from anti-TNFα (18). This suggests that the pathogenic mechanisms induced by bacterial infection are much more complicated than a cytokine storm, and strongly support the involvement of other immune response mechanisms (20). Nevertheless, the applicability of these models to human diseases and their influence on the designing novel therapeutics continue to be subjects of discussion (21, 22).

Besides LPS and CLP, other animal models have been established (23, 24) to delve into the mechanisms that underline gut-injury-induced systemic inflammation. They notably aim to elucidate two critical facets: the resultant epithelial damage and the emulation of luminal bacterial infection. The cecal slurry injection model (23, 25), for example, introduces a fecal slurry into the peritoneal cavity, triggering the release of bacterial products and initiating a cascade of pro-inflammatory cytokines (26). Another model of significance is the intra-abdominal abscess model (27), which simulates clinical scenarios of intra-abdominal infections and provides vital insights into the event sequence culminating in lethal inflammation (28). Models incorporating ischemia-reperfusion and burn injuries in rodents have enhanced our comprehension by identifying the complex mechanisms through which gut-barrier malfunctions instigate systemic inflammation (29). The activation of particular molecular pathways, such as NF-κB and IL1β production in gut injuries, has been emphasized in this context (30). However, the quest for an ideal model that accurately replicates the clinical transition from localized gut injury to potentially life-threatening systemic manifestations still sustains (31).

The Dextran Sulfate Sodium (DSS) model, commonly used to induce experimental colitis in rodents (32, 33), shows significant promise. A unique characteristic of the DSS model is its ability to induce colonic damage and acute inflammation lasting 5-7 days, offering a longer duration for mechanistic studies, especially in myeloid cell function. It’s essential to understand that the effectiveness of DSS in causing colitis can be affected by factors like the genetic background of the mice, and the source and dosage of DSS. In past research, we used a 2%-2.5% DSS concentration, consistently producing standard colitis symptoms in C57BL/6J wild-type mice (33). This study reveals that a higher DSS concentration, such as 4%, can constantly exacerbate colon injuries and might induce lethal systemic inflammation, making it a valuable model for gut-systemic inflammation research. However, the effects of this pronounced injury on distant organs require more exploration.

The spleen, a major lymphoid organ, plays a pivotal role in gut-induced systemic inflammation, acting as the primary filter for blood pathogens and regulating erythrocyte balance (34). Its anatomical position within the systemic circulation allows it to act as a central point for the immune system, recruiting and activating immune cells like macrophages and T cells when inflammatory signals are detected (35). When the gastrointestinal barrier is breached, bacteria and toxins, notably lipopolysaccharides (LPS), enter the bloodstream and trigger spleen reactions (36, 37). Recent studies revealed that mice undergoing splenectomy exhibited more pronounced mucositis when exposed to DSS compared to sham-DSS mice, as evidenced by factors like mortality rate, stool consistency, gut barrier defects, serum cytokine levels, and blood bacterial counts. Notably, the detection of green fluorescent-producing *E. coli* in the spleen of DSS mice post-oral gavage underscores the spleen’s vital role in managing gut bacterial translocation (38, 39). This suggests that gut dysbiosis and bacterial translocation in splenectomy patients could link to systemic infections. Moreover, a compromised spleen’s ability to filter damaged red blood cells can escalate inflammation, risking multi-organ failure (40). A deeper understanding of the connection between spleen functionality and systemic inflammation is needed.

In our study, we developed a 4% DSS-induced severe colon-injury animal model to elucidate the broader implications of localized colonic inflammation. Unlike traditional DSS models, our approach allowed limited recovery time. Utilizing techniques including histology, *in situ* hybridization, flow cytometry, and the Seahorse assay, we meticulously examined systemic inflammatory markers, identified injury patterns in the liver and lungs, and conducted a thorough analysis of spleen characteristics. Our data revealed (1): Elevated DSS concentrations amplified colonic damage, leading to increased mortality (2). Pronounced bacterial infiltration in the muscularis layers resulted in marked peripheral inflammation, as observed in hepatic and pulmonary injuries (3). Observations in the spleen indicated diminished metabolic activity, decreased CD45 expression, and increased splenocyte apoptosis (4). A review of the human inflammatory bowel disease (IBD) database underscored the increase in systemic inflammatory markers correlated with intestinal damage. Collectively, these findings suggest that severe intestinal inflammation may facilitate bacterial translocation and induce systemic immune responses, providing insights for therapeutic strategies targeting systemic inflammation arising from intestinal injury and infection.

## Results

### High-dose 4% DSS induced severe colonic damage and lethal inflammation in mice

**Figure 1:**
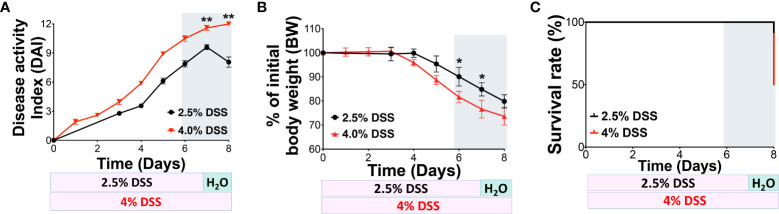
4% DSS increased the DAI score and mortality in C57BL/6J mice. **(A)** Comparison of 2.5% and 4% DSS-induced disease in mice, as indicated by the Disease Activity Index (DAI) score. The DAI score, reflecting disease severity, was calculated daily based on three parameters: weight loss, stool consistency, and rectal bleeding. Each parameter was scored from 0 to 4, with the cumulative score ranging from 0 to 12; 12 represents the maximum severity of colon injury. **(B)** Changes in body weight (BW) were monitored daily and used as an indicator of disease progression. **(C)** The daily survival rate was recorded for both treatment groups. The data presented are representative of two independent experiments and are shown as the mean ± standard error of the mean (s.e.m.). Groups were statistically compared using a two-tailed Student’s t-test, with n = 6 per group; *p < 0.05, **p < 0.01, indicating statistical significance.

To determine if severe gut barrier dysfunction can lead to systemic inflammation, we established a model using DSS-induced intestinal injury. Initially, we contrasted two distinct protocols. C57/BL-6J mice were administered either 2.5% DSS or 4% DSS drinking water (from Alfa Aesar, J63606) as described in **Figure 1**. We observed the animals for body weight changes, Disease-Associated Index (DAI), and survival rates. The 2.5% DSS administration led to an average DAI of 8 and a decline in body weight by 10-12% on day 7. In comparison, mice subjected to the high-dose DSS presented a DAI of 12 and a body weight reduction of 20-25% on day 7 (**Figures 1A, B**). In line with prior observations, the 2.5% DSS-colitis model showed no significant mortality during the acute phase (DSS days 5-7), stabilizing at a survival rate of 85%-90% by day 8. However, the high-dose 4% DSS group had markedly reduced survival rates, reaching 50% by day 8 (**Figure 1C**). These findings highlight the differences in outcomes between the high-dose 4% DSS and low-dose 2.5% DSS treatments, considering parameters like DAI, body weight, and survival rate, which correlate with disease progression.

### High-dose 4% DSS disrupted colon barrier integrity, leading to bacterial penetration and systemic inflammation

**Figure 2:**
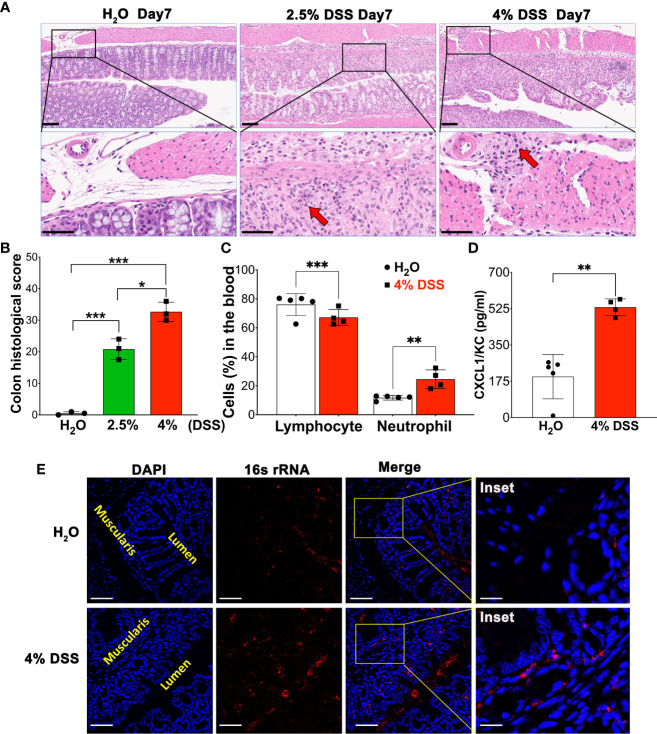
4% DSS severely disrupted colon barrier integrity, leading to bacterial translocation and peripheral inflammation. **(A)** A representative image illustrating the comparison of colon injury severity under homeostatic conditions, 2.5% DSS treatment, and 4% DSS treatment. On day 7, colon samples from various conditions were subjected to H&E staining. Severity was scored from 0 to 3, based on criteria such as epithelial injury and infiltration into the mucosal, submucosal, and muscularis layers (details in **Table 1**). These are representative images from three independent experiments. Scale bars - Upper panel: 100 µm; Lower panel: 50 µm. **(B)** Depiction of the assessed severity of epithelial damage and the extent of inflammatory infiltration in panel A, with values presented as mean ± s.e.m. Statistical significance was determined using one-way ANOVA, with *p < 0.05, ***p < 0.001, and each group comprising three samples. **(C)** The Complete Blood Count (CBC) values for both vehicle-treated animals (n=5) and 4% DSS-treated animals (n=4). Results are expressed as mean ± s.e.m, with statistical analysis performed using an unpaired two-tailed Student’s t-test, **p < 0.01, ***p < 0.001, representative of two independent experiments. **(D)** Quantification of the inflammatory mediator KC/CXCL-1 in blood serum using Bio-Plex. The data are reported as mean ± s.e.m. Statistical significance was assessed with an unpaired two-tailed Student’s t-test, **p < 0.01, representative of two independent experiments. **(E)** Gut bacterial localization in the colon, comparing control (H_2_O group) with the 4% DSS-induced colitis model, was visualized using RNAScope assay. The Fluorescence *in situ* hybridization (FISH) technique included hybridization with a 16S rRNA probe and subsequent signal amplification using TSA-Cy3 (red), allowing for the visualization of gut bacteria on day 7 post-induction. Notably, in the 4% DSS group, the presence of red dots within the muscularis compartment indicates bacterial penetration, a stark contrast to the H_2_O group where bacteria remain confined to the lumen. The lumen and muscularis compartments are highlighted in yellow, scale bars - 100 µm and 25 µm (inset). These images are representative of two independent experiments, with n = 4 mice per group.

In this study, we emphasized the severity of gut mucosa damage and determined systemic inflammation parameters using a 4% DSS model. We collected colonic tissues on day 7 and assessed DSS-induced colonic mucosal injury using Hematoxylin & Eosin (H&E) staining (**Figure 2A**). On day 7, we observed that a high dose of DSS significantly compromised colonic barrier integrity. This was evidenced by severe erosion of the epithelial monolayer and extensive immune cell infiltration into the muscularis compared to both the vehicle and the 2.5% DSS group, as presented in **Figure 2A**. The histologic changes observed were determined blindly, and the average scores for the different groups were calculated and illustrated in **Figure 2B**. Moreover, we evaluated the complete blood count (CBC) and observed a decline in lymphocytes, while neutrophils increased as the disease progressed (**Figure 2C**). To further elucidate the link between pronounced gut mucosal damage and systemic inflammation, we employed Bio-plex to measure serum concentrations of the chemokine CXCL1/KC, which is recognized for its elevated presence and ability to attract neutrophils during systemic inflammation. Notably, we observed a significant increase in CXCR1/KC levels in the serum of the high-dose group on day 7 (**Figure 2D**). Finally, using a 16S bacterial RNA probe and RNAScope technology, we performed fluorescence *in situ* hybridization (FISH) to track bacterial penetration from the mucosal surface to deeper compartment of the colon. Our data unveiled those colonic bacteria had migrated to the muscularis/serosa epithelium (**Figure 2E**). Together, our findings suggest that extended and high-dose DSS treatment compromises the colon barrier, fosters the migration of gut bacteria, and could potentially intensify systemic inflammation.

### High-dose 4% DSS animals showed distal organ injury and systemic inflammation

**Figure 3:**
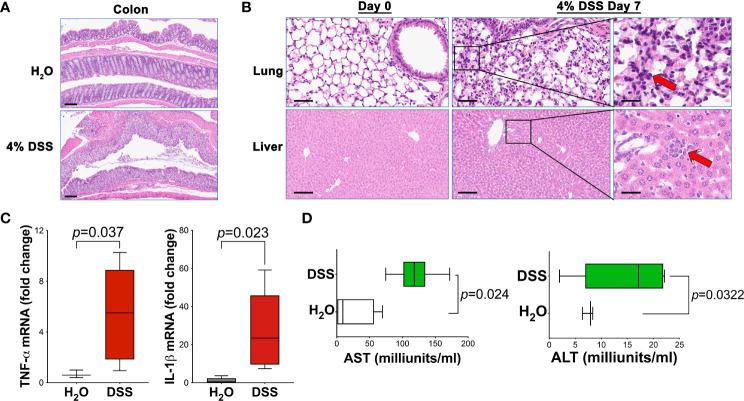
4% DSS induced distal tissue injury and systemic inflammation. **(A)** Histological assessment of colon injury in animals treated with either vehicle H_2_O or 4% DSS, Scale bar - 100 µm. **(B)** Liver and lung samples from both H_2_O vehicle and 4% DSS groups were collected, fixed in formalin for 16 hours, and then processed for paraffin embedding, sectioning, and H&E staining. Representative images of liver and lung tissues are shown before (day 0) and after (day 7) DSS treatment. Red arrows highlight the infiltrating inflammatory cells (n = 3 per group), Scale bars - 200 µm and 50 µm (inset). **(C)** The expression levels of pro-inflammatory cytokines *Tnfa* and *Il1b* in liver tissue were quantified using real-time PCR. mRNA was extracted from liver samples of the study animals, and reverse transcription-quantitative PCR (RT-qPCR) was conducted to evaluate the expression of these specific genes. Results are expressed as mean ± s.e.m. Statistical significance *p* value was determined using an unpaired two-tailed Student’s t-test. The results are representative of two independent experiments. **(D)** ALT and AST assays were conducted to assess the serum concentrations of alanine transaminase (ALT) and aspartate transaminase (AST) at day 7 post-DSS treatment, using the H_2_O vehicle as a control. The results are representative of two combined experiments. The ALT or AST activity (U/L) is calculated as: (ΔA/min × Total assay volume × Dilution factor)/(6.22 × Sample volume). Statistical significance was assessed with an unpaired two-tailed Student’s t-test. Results are expressed as mean ± s.e.m. Data were combined from two separate studies, each with four to six mice in every group for each experiment.

Given the elevated mortality rates and systemic inflammation symptoms in 4% of DSS animals, we sought to examine the possible link between significant gut mucosal damage and subsequent effects on distal organs. We hypothesized that severe gut damage (**Figure 3A**), without prompt wound healing, could induce systemic inflammation and injure distant organs, such as the liver and lungs. Histological analysis using HE stain revealed immune cell infiltration in the lungs and liver following high-dose DSS administration (**Figure 3B**), strongly suggesting that gut dysfunction precipitated disease progression to distal tissues, indicative of systemic inflammation.

To corroborate these findings, we analyzed the expression of pro-inflammatory cytokines in the liver using real-time PCR. As anticipated, DSS animals demonstrated a significant overexpression of specific pro-inflammatory cytokines, namely *Il1b* and *Tnfa* **Figure 3C**). This elevated cytokine expression, crucial for indicating ongoing inflammation, further substantiates the concept that systemic inflammation is instigated by gut injury. Additionally, we evaluated the liver injury markers, Alanine transaminase (ALT) and Aspartate transaminase (AST), in animal serum. Our findings showed an increased level of these markers in the serum of mice treated with a high dose of DSS (**Figure 3D**). This suggests that the liver function may be compromised, likely resulting from the systemic inflammatory response initiated by gut injury.

Collectively, our findings depicted in **Figure 3** affirm that gut barrier dysfunction can result in injury to distal tissues and foster the evolution of lethal systemic inflammation. The noted immune cell infiltration in the lungs and liver, alongside the upregulation of pro-inflammatory cytokines and liver injury markers, cohesively illustrates the detrimental progression of gut-originated inflammation to distally affect organs.

### Metabolic repression and apoptosis in splenocytes linked to gut injury-induced systemic inflammation

**Figure 4:**
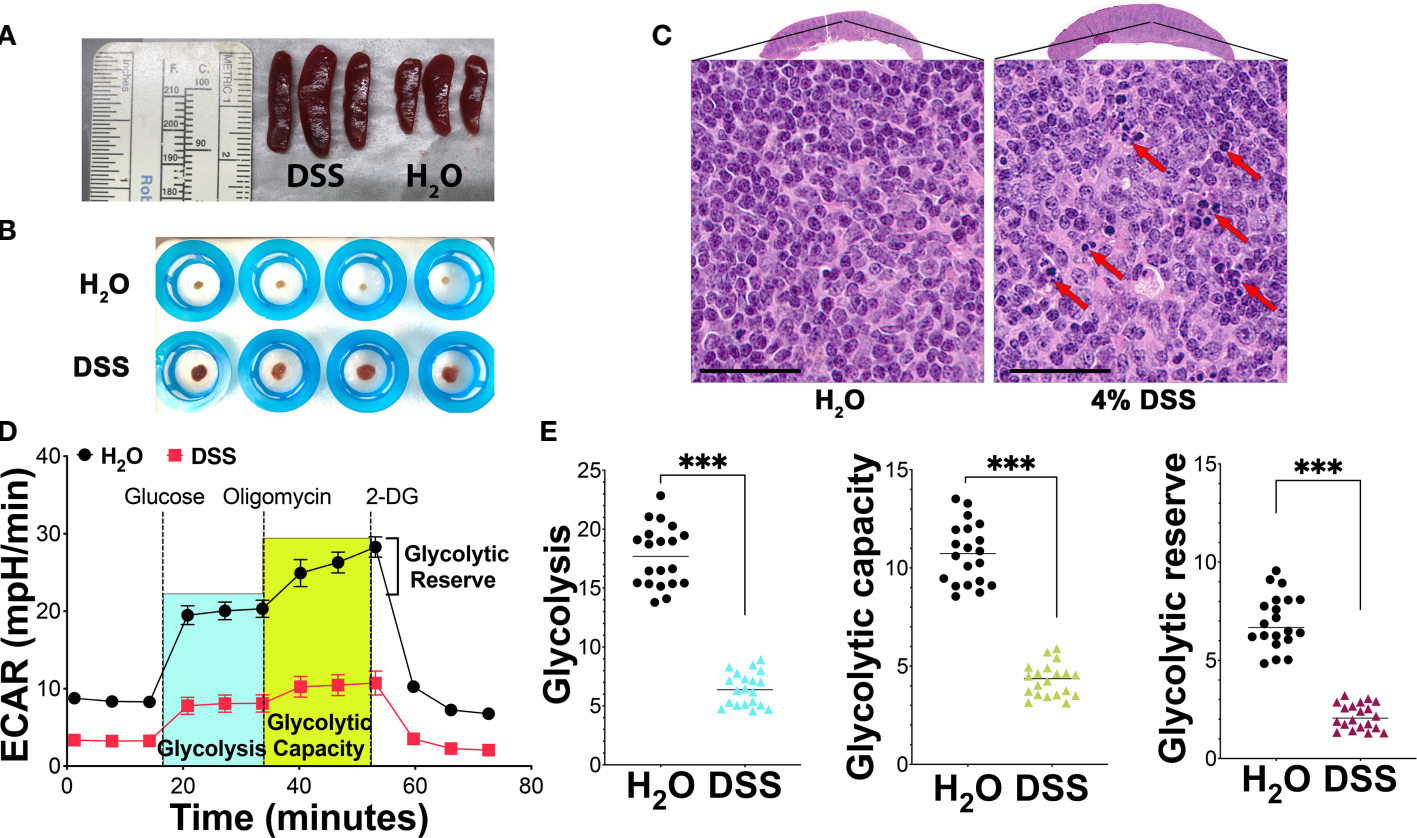
Metabolic suppression and apoptosis in splenocytes during mucosal injury-induced systemic inflammation. **(A)** Spleen volume analysis: A significant increase in spleen volume was noted in mice treated with 4% DSS by day 7 in comparison to the H_2_O vehicle-treated control group, with each group consisting of three mice. The data is representative of three separate experiments. **(B)** Mesenchymal component analysis: This analysis compared the mesenchymal component of the spleen between the H_2_O vehicle group and the 4% DSS-treated group, with four mice in each group. The results presented are from a series of three independent experiments. **(C)** Spleen morphology and apoptosis assessment: HE-stained spleen sections from mice treated with 4% DSS show distinct morphological changes, scale bar - 50 µm. Apoptotic cells, indicated by red arrows, were observed both individually and in clusters. These images are selected from two different experimental runs, with each group containing four mice. **(D)** Glycolytic activity via Seahorse Assay: Glycolytic activity was evaluated using the Agilent Seahorse XF Glycolysis Stress Test on 300,000 splenocytes per well, plated on a poly-D-lysine (PDL)-coated plate. The extracellular acidification rate (ECAR) was recorded over time at specified intervals of the assay, with values presented as mean ± s.e.m. **(E)** Quantitative glycolysis analysis: This analysis quantified glycolytic function, capacity, and reserve in splenocytes from both H_2_O and 4% DSS-treated groups as described in panel **(D)**. The statistical significance was assessed using the Student’s t-test, with ***p<0.001 indicating high significance. These results are derived from two independent experiments. Each experiment included biological replicates, consisting of four mice per group, and technical replicates, with five replicated wells per mouse.

The spleen, vital in regulating immune responses and filtering pathogens during systemic inflammation (41), exhibited notable changes in the presence of gut injury-associated systemic inflammation in our study. In mice treated with 4% DSS, spleen volume increased to approximately 1.5-2 times that of controls (**Figure 4A**), with an elevated mesenchymal component observed in the spleens of high-dose DSS animals (**Figure 4B**). Moreover, in our spleen histologic analysis using HE stain, we observed that apoptosis affects individual cells or small cell clusters. The apoptotic cells are characterized by their round or oval shape, dark eosinophilic cytoplasm, and dense purple nuclear chromatin fragments. (**Figure 4C**). These findings suggest that mucosal barrier dysfunction gives rise to altered spleen phenotypes. Given the known association between splenic immune cell metabolism and function (42), we utilized the Seahorse assay to explore how splenic immunosuppression might influence the overarching inflammatory process.

We focused on glycolysis, a principal cellular metabolism pathway and crucial energy source during disease pathogenesis. Employing a glycolytic stress kit to evaluate the capacity of the glycolytic pathway post-glucose starvation, we first observed no notable difference in the glycolysis baseline between the vehicle group and the gut injury-induced systemic inflammation group (**Figure 4C**). However, deeper analysis revealed a significant disparity in splenocyte glycolysis, glycolytic capacity, and glycolytic reserve between the two groups. In the group treated with 4% DSS, there was a marked decrease in glycolysis, pointing to compromised energy production in the spleen’s immune cells. This treatment also led to a reduced glycolytic capacity, the maximum potential for glycolysis to generate ATP, as illustrated in **Figures 4D, E**. Subsequent analysis showed that the high-dose DSS animals had about a 30% decline in their glycolytic reserve, a measure of a cell’s ability to boost glycolysis under increased energy demands, in comparison to the control group (**Figures 4D, E**). This reduced reserve suggests that the splenic immune cells may have limited capacity to meet heightened metabolic requirements during systemic inflammatory stress. In alignment with the Seahorse assay data, splenocytes displayed pronounced metabolic repression in glycolytic activity, indicating that the inhibition of splenocyte metabolism during systemic inflammatory stress potentially damages the immune cell function of the spleen.

### Splenocyte apoptosis associated with immune cell dysfunction in gut injury-associated systemic inflammation

**Figure 5:**
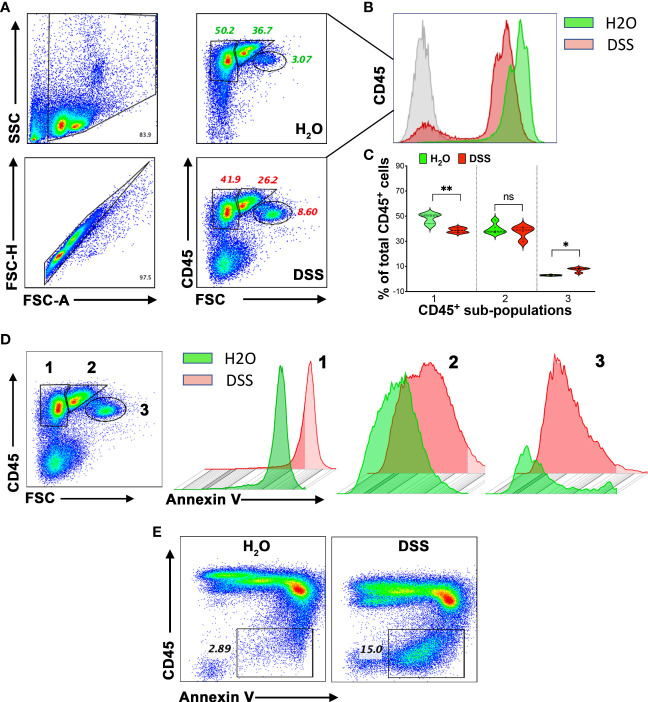
Apoptosis connected to splenic immune cell dysfunction in the gut-injury-associated inflammation. **(A)** CD45 expression in Splenocytes. Comparative study between the H_2_O and 4% DSS groups. Splenocytes, harvested on day 7, were subjected to flow cytometry for CD45 expression, revealing three distinct subpopulations. (n=4 mice/group). **(B)** CD45 marker decline. Notable decrease in CD45 expression in the spleens from the 4% DSS-treated animals. (n=4 mice/group). **(C)** Quantitative analysis of CD45^+^ spleen subpopulations. This figure presents the relative proportions of different CD45^+^ cell subpopulations, normalized to the H_2_O control group. Statistical significance was calculated using one-way ANOVA, with *p < 0.05, **p < 0.01, and ns denoting not significant. **(D)** Annexin V expression in CD45^+^ subpopulations. Analysis across CD45^+^ subsets, with data visualized in both scatter plots and histograms. (n=4 mice/group). **(E)** Flow Cytometric Analysis of CD45 and Annexin V. Using flow cytometry, CD45 and Annexin V were detected. Following high-dose DSS treatment on day 7, a specific splenocyte population exhibiting apoptosis emerged and lacked the CD45 marker. These results represent data from two independent experiments, with four mice per group. The Q-Q plot validated the data set’s normality before the statistical analysis.

Given the known association between increased splenic immune cell death, bacterial distribution, and elevated pro-inflammatory cytokines produced by the host (43), we hypothesized that splenocyte immunosuppression in animals might be driven by amplified immune cell apoptosis. To further evaluate the status of immune cells and the observation of apoptosis in the spleen (**Figure 4C**), we isolated these cells and utilized CD45 and Annexin V staining for flow cytometry analysis. Firstly, we found a general decrease in the CD45 marker in the 4% DSS group (**Figures 5A, B**), potentially indicating either a diminished white blood cell presence or compromised T cell function, as CD45 is a transmembrane tyrosine phosphatase involved in T cell receptor signaling and T cell development (44). Further, examination of CD45^-high^ cells elucidated three distinct populations, each with varying cell numbers (**Figures 5A, C**). We then turned our attention to identifying apoptosis within specific CD45-high subpopulations. Notably, we discerned a statistically significant increase in Annexin V-positive cells in subpopulation 3, which also demonstrated relatively lower CD45 expression compared to subpopulations 1 and 2 (**Figure 5D**). Interestingly, a novel population, characterized by the loss of the CD45 marker and enrichment of Annexin V-positive cells, emerged in the high-dose DSS animals (**Figure 5E**). This discovery is particularly noteworthy as it indicates a correlation between immune cell apoptosis and compromised function within the spleen. Taken together, our results indicate that the heightened apoptosis observed in splenic immune cells may contribute to the elevated levels of pro-inflammatory cytokines detected in the circulation in the gut-injury systemic inflammation model. Furthermore, the presence of CD45-negative cells and apoptosis in the spleen implies their potential involvement in spleen dysfunction and the advancement of lethal systemic inflammation associated with gut injury.

### Bioinformatics analysis in humans showed that IBD patients with severe gut injury are associated with systemic inflammation

**Figure 6:**
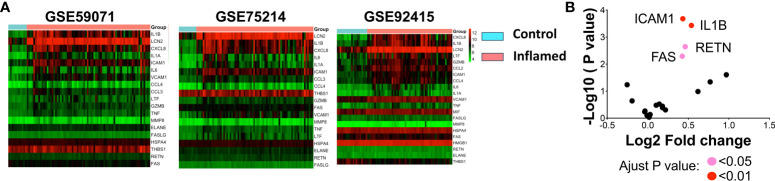
Bioinformatic analysis of systemic inflammation-associated genes in IBD databases. **(A)** Biomarkers taken from various panels of the human sepsis Bio-Plex detection assay (including IL1ß, LCN2, CXCL8, IL6, IL1A, ICM1, CCL3, CCL4, THBS1, GZMB, FAS, VCAM1, MMP8, TNF, LIF, HSPA4, ELANE, RETN, and FASLG) were analyzed using human IBD colon biopsy databases. Changes in gene expression within the colon were calculated, and log2 fold-change (FC) values were utilized to create a heatmap. Red signifies increased expression in inflamed samples compared to controls, while green indicates decreased expression. **(B)** Significant changes in blood gene expression are depicted in a volcano plot sourced from the GSE92415 database. A pink dot indicates an adjusted *p* < 0.05 for genes *FAS* and *RETN*, and a red dot reveals an adjusted *p* < 0.01 for genes *ICAM1* and *IL1B*. Note: Databases GSE59071, GSE75214, and GSE92415 are human microarray databases.

To corroborate our hypothesis— that severe gut injury induces lethal systemic inflammation— we studied its correlation by exploring the Inflammatory Bowel Disease (IBD) patient database. Utilizing three human microarray databases (GSE59071, GSE75214, and GSE92415), we investigated systemic inflammatory markers in IBD patients, revealing a notable link between gut injury and the upregulation of certain markers. We selected these biomarkers from the human Bio-multiplex sepsis panels, which include IL1B, LCN2, CXCL8, IL6, IL1A, ICAM1, CCL3, CCL4, THBS1, GZMB, FAS, VCAM1, MMP8, TNF, LIF, HSPA4, ELANE, RETN, and FASLG. Consistently across the three colon biopsy databases, we identified the upregulation of *IL1B, CXCL8, and LCN2* in IBD patients, which is visually represented in a heatmap (**Figure 6A**). Moreover, we found *ICAM1, IL1B, FAS, and RETN* to be statistically elevated in the blood of these IBD patients (**Figure 6B**). This implies that gut inflammation influences not just the local compartment, but also instigates systemic responses, evidenced by an increase in circulating septic markers. Consequently, our findings underscore a possible connection between gut health and systemic inflammation in IBD patients, emphasizing the necessity to comprehend the broader impacts of gut injury and inflammation beyond merely the localized context.

## Discussion

Intestinal injury and lethal systemic inflammation demonstrate a bidirectional relationship, as evidenced by the increased risk of sepsis in patients with gut infections or injuries and the frequent presence of intestinal damage in those with sepsis (45) This interplay complicates pathogenesis and poses challenges to accurately replicating the progression in animal models. Numerous animal models have been developed to emulate various clinical scenarios (46). In this study, using a high-dose DSS-induced gut injury model, we investigated systemic inflammation development and delved into the potential mechanisms during this process. Our findings not only identified severe gut injury developing into lethal inflammation but also unveiled the splenocyte dysfunction associated with the pathogenesis. Further, Bioinformatics analysis of human IBD patient data corroborated our findings, underscoring the clinical relevance to gut injury-associated systemic inflammation.

For decades, LPS, CLP, and various other animal models have been utilized to explore systemic inflammation, sepsis, and multiple organ dysfunction (MOD). While each of these models provides its own set of advantages and limitations, they have all played a role in identifying crucial disease mechanisms. However, despite their contributions, the therapeutic strategies and clinical outcomes derived from these studies remain suboptimal. This underscores the pressing need for a more refined research model. The DSS model, replicating acute colitis, is extensively utilized in research on epithelial damage and wound healing. Recent studies indicate that DSS-induced mucosal injuries enhance epithelial permeability, raising the infection and developing into a lethal sepsis-like disease (15). Using both high and low-dose DSS models, we noted varied symptoms and outcomes. Animals subjected to a high dose of DSS exhibited not only significant weight loss, elevated DAI scores, and increased mortality but also profound mucosal damage. Furthermore, there was a rise in pro-inflammatory cytokines in the bloodstream and an influx of immune cells in both the colon and distant tissues. These findings highlight the link between localized colon injury and systemic immune responses.

Our high-dose DSS model offers several advantages over previous models (1). Extended acute phase for mechanism study: While traditional models such as CLP and LPS rapidly develop symptoms, they might not encompass the intricacies of bacterial infections beyond cytokine storms. In contrast, our model has a 5- to 6-day acute phase before the onset of lethal inflammation. This feature is invaluable for delving into intricate pathogenic processes, offering a broader timeframe for comprehensive study (2). Focus on innate immunity: Lethal inflammation or sepsis initially involves the innate immune system (47). Our DSS model triggers inflammation primarily through innate immune components, especially monocytes and macrophages (Mφ) (48), making it an ideal framework for investigating innate immunity in-depth (3). Clinical relevance: The DSS model holds significant relevance to human conditions, particularly those involving mucosal damage or sepsis following intestinal surgery. This connection to real-world medical scenarios enhances the applicability and value of the research conducted using this model (4). Consistency and reliability: While the CLP model is commonly used, it has variability issues and doesn’t always induce lung injury (18). In contrast, our high-dose DSS model is simpler, surgery-free, and consistently shows a connection between mucosal injury events and systemic inflammation development (49). In sum, our model, based on the DSS-induced acute colitis approach, offers a reliable, clinically relevant, and innate immunity-focused framework for lethal systemic inflammation research, addressing the limitations of existing models.

Our current model, based on the C57BL/6J mouse strain, has its limitations. Its exclusivity raises concerns about its broader applicability in other strains. Additionally, we’ve only focused on day 7 due to high mortality rates observed between days 8 to 10, which might overlook dynamic inflammatory changes over time. To navigate these limitations in future research, we plan to utilize various animal strains, explore additional intestinal injury causes, investigate different time points, and evaluate the efficacy of potential therapeutic interventions, thereby fostering a logical and comprehensive approach. Notably, we didn’t delve into risk factors like antibiotic usage and gut microbiome imbalances in our current study. These represent vital aspects that need exploration. Despite acknowledging intestinal barrier defects and microbiome dysbiosis as major sepsis risk factors, their clinical implementation remains limited, a challenge we aim to address in our refined model. In addition, patients with severe sepsis often exhibit dysfunction in the heart and kidneys (50, 51). Currently, a suitable rodent model to investigate these pathophysiological consequences is lacking. It would be intriguing to determine if our newly developed severe gut injury-induced sepsis model can be applied to study the pathophysiology of sepsis-associated dysfunction in the cardiovascular and renal systems in future research.

The colon, when inflamed, can impact systemic inflammation through its role in the gut-lymphatic pathway. Lymphoid organs, which include the lymph nodes, spleen, and bone marrow, are key in orchestrating immune responses and may become sites of excessive immune cell activation or dysfunction during such inflammation. This interaction can contribute to the pathogenesis of gut-associated systemic inflammation. Notably, the spleen often exhibits the earliest detectable changes due to its visibility and pronounced response, suggesting a potentially primary role of lymphoid organs in the pathology of systemic diseases. As the largest lymphoid organ, the spleen plays a critical role in monitoring blood and responding to pathogens. Within the spleen are various leukocytes, such as T and B cells, dendritic cells (DCs), and macrophages, fundamental for maintaining homeostasis and mounting immune responses in situations like sepsis (34, 52). Recent study suggest that dysfunction in the spleen’s immune cells can precipitate systemic inflammation, sepsis, and multiple organ dysfunction syndrome (MODS) through various mechanisms (34). One mechanism involves the disruption of T-cell homeostasis by septic impact. In our model, although the specific apoptotic cell type was not identified, we hypothesize that T-cells play a crucial role in severe systemic inflammation, significantly influencing the observed spleen phenotype. Prior research indicates that sepsis prompts apoptosis in immune cells, especially in the spleen, resulting in a marked reduction of CD4^+^ and CD8^+^ T cells. This apoptotic activity is a key factor in the immunosuppressive phase of sepsis. Regulatory T cells appear to be more resilient to this apoptotic effect. However, the CD4^+^ and CD8^+^ T cells that do survive often adopt a less effective T cell function or enter a state of ‘exhaustion’ that compromises the immune response. Strategies that target the apoptosis of T cells and block the PD-1 and PD-L1 pathways are being explored as potential methods to counteract T cell dysfunction and thereby improve sepsis outcomes (53, 54). Furthermore, immunometabolism influences the behavior and functionality of immune cells. For instance, there’s a metabolic shift observed in spleen Treg cells in septic conditions (55). Supporting this, a study indicated that IFNγ enhances glycolysis via the PI3K/Akt/mTOR pathway, which potentially counters spleen immunosuppression during sepsis (56). In addition, CD45, an essential transmembrane glycoprotein, is pivotal for immune functionality. It aids TCR signaling by activating Lck, a key component of the TCR complex (57). The importance of CD45 is further emphasized by studies on CD45 mutants and deficient organisms (58, 59). Mechanically, recent evidence suggests that heightened intestinal permeability correlates with an increase in apoptotic splenocytes, which intensifies sepsis (15). In our experiments, observed spleen enlargement and intensified mesenchymal content could suggest splenic malfunction. It’s noteworthy that glycolysis converts glucose to energy. Our findings of decreased glycolysis in the spleen might relate to systemic inflammation’s lethality, it underscores the significance of investigating splenic immune cell metabolism, echoing earlier research on splenocyte metabolic reprogramming (56). The abundance of Annexin V-rich apoptotic cells among splenocytes with diminished CD45 levels in our study might shed light on the immunosuppressive characteristics observed and their link to the onset of lethal systemic inflammation. Yet, conclusions should be drawn cautiously due to no evaluation of other mitochondrial pathways and the absence of a loss-of-function assay.

To translate our findings into clinical practice, we analyzed three human microarray databases, enabling us to validate specific systemic inflammation markers in IBD patients experiencing gut injury and inflammation. By comparing 20 biomarkers across three human septic panels, we ascertained that *ICAM1, IL1B, FAS, and RETN* were statistically elevated in the blood of IBD patients, which indicates the existence of a gut injury-induced systemic inflammation pathway. However, this analysis does present certain limitations. First, the severity of gut injury in the patients was not thoroughly documented, which could influence the observed results. Moreover, the utilization of microarray data in all three databases, as opposed to deep sequencing, limits the depth of insight into transcriptome-level changes.

**Figure 7:**
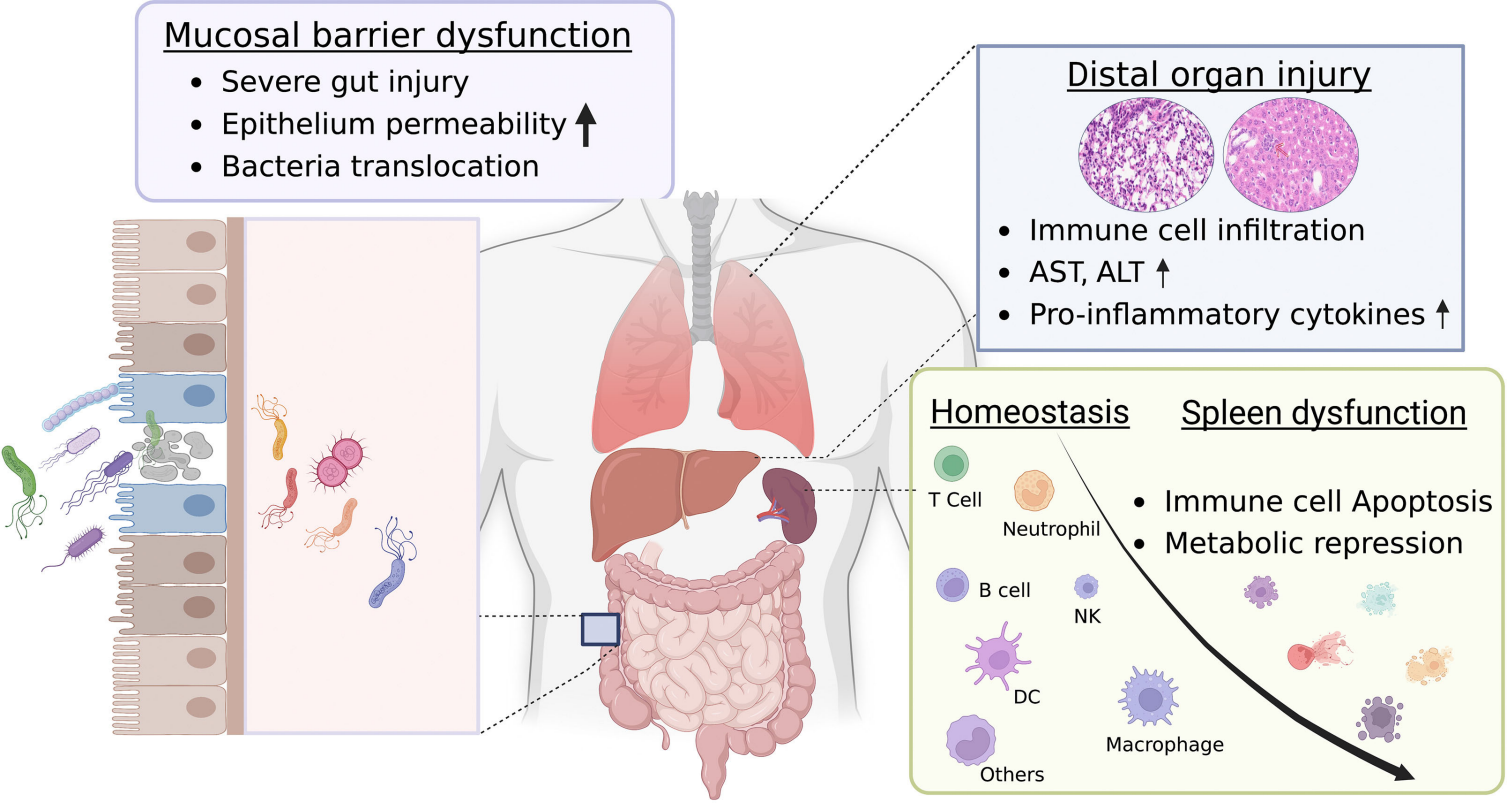
Proposed graphic mechanism of gut-associated systemic inflammation. The gut plays a crucial role in maintaining overall health. Dysfunction of the gut can result in systemic inflammation, which can have potentially severe consequences. The intestinal barrier, which consists of a single layer of epithelial cells bound together by tight junctions, regulates the movement of molecules. This allows for the absorption of nutrients while preventing harmful substances from entering the bloodstream. However, when these tight junctions are damaged or weakened, a condition termed “gut leakage” arises. This leads to the entry of harmful entities such as bacteria, toxins, and undigested food particles into the bloodstream, subsequently triggering gut-associated systemic inflammation and injury to distal organs. While the link between gut leakage and inflammation is acknowledged, the exact mechanism underlying this relationship remains elusive. Dysfunction of the spleen, characterized by metabolic reprogramming and apoptosis of immune cells, has been pinpointed as a key contributor to the onset and worsening of systemic inflammation. The reduced functionality of the spleen hinders its capability to effectively filter and eliminate harmful substances that have entered the bloodstream due to gut leakage. As a result, this disruption intensifies the systemic inflammatory response, creating a vicious cycle of inflammation and organ damage. Recognizing the crucial role of spleen dysfunction in gut-associated systemic inflammation can pave the way for targeted interventions and treatments, potentially reducing the impact of inflammatory conditions and enhancing overall health outcomes. This figure was created with license from Biorender.

In conclusion, our high-dose DSS mucosal injury model highlights the development from gut injury to severe systemic inflammation. We found that severe mucosal damage promotes bacterial movement to the muscularis compartment, leading to peripheral inflammation and distal organ damage. Insights into metabolic changes, a decrease in the CD45 marker, and an increase in splenocyte apoptosis potentially offer an association with pathogenesis, which is valuable for sepsis research. As illustrated in **Figure 7**, mucosal injury acts as a primary stimulus for systemic inflammation. This inflammation is influenced by risk factors such as oxidative stress, bacterial infections, and gut microbiome dysbiosis. Consequently, this injury weakens intestinal barriers, leading to increased permeability. Any leakage activates the immune system, which, if extreme, can cause a “cytokine storm”, leading to multi-organ damage and severe systemic inflammation. A deeper grasp of these processes is essential for developing new treatments. Our model seeks to fill the knowledge gap in relevant animal models. Our insights expand the DSS model’s applicability in exploring the relationship between severe intestinal injuries and lethal inflammation, deepening our comprehension of its pathogenesis.

## Methods

### Animal model

C57BL/6 WT mice were originally ordered from the Jackson Laboratory (JAX cat no. 000664) and employed in our research. All mice were housed in a temperature-controlled specific pathogen-free Animal Care Center (ACC) at the University of Illinois at Chicago. All animal experiments were conducted under the National Institute of Health guidelines for the Care and Use of Laboratory Animals and were approved by the IACUC. By modifying the classical DSS-colitis model, we developed a DSS-systemic inflammation model utilizing C57BL/6 WT mice aged between 8 and 12 weeks. Preliminary experiments suggested a DSS concentration ranging from 2.0% to 2.5% (prepared w/v in sterile water and offered ad libitum) resulting in DSS colitis. Based on this observation, we determined to use 4% DSS to induce systemic inflammation in our tailored model. For the DSS treatment (molecular mass 40,000; Alfa Acesar), we adhered to our previously established protocol (33). Adult mice were administered varying doses of DSS for up to 7 days. This was followed by regular drinking water in the DSS-colitis model or an additional day of 4% DSS treatment in the DSS-systemic inflammation model. The DSS solutions were made freshly every two days. At the end of the experiments, the animals were euthanized with CO2 inhalation followed by cervical dislocation.

#### Disease activity index and histological scoring in the DSS model

The severity of DSS-induced colon injury was assessed daily using the Disease Activity Index (DAI), which takes into consideration factors such as body weight loss, stool consistency, and the severity of fecal occult blood tests (33). Additionally, histological scoring was performed using hematoxylin and eosin (H&E) staining. In brief, the colon was measured, fixed in neutral buffered formalin (NBF) for 16 hours, embedded in paraffin, and sectioned into 5 μm thickness slices, which were then stained with H&E. The assessment was conducted blindly by a trained gastroenteric pathologist who assigned scores of 0-3 to each section, reflecting the extent of epithelial damage and the intensity of inflammatory infiltration in various regions, including the mucosa, submucosa, and muscularis/serosa. Depending on the nature of observed changes, whether they were focal, patchy, or diffuse, the scores were multiplied by factors of 1, 2, or 3, respectively. Cumulative scores from individual sections were then tabulated, resulting in a potential score range of 0-36 per mouse (60) (Please refer to **Table 1** for more details).

**Table 1:** Histology scoring for severity of colon injury.

Histological Hematoxylin & Eosin		H_2_O	2.5%-DSS	4%-DSS
Epithelial damage		0	1.5	3
Inflammatory infiltration	Mucosa	0	2	3
	Submucosa	0	1	3
	Muscularis/serosa	0	0	2

Each of the four scores is multiplied by 1 if the change is focal, 2 if it is patchy, and 3 if it is diffuse.

#### 16s rRNA *in situ* hybridization

RNAScope is a patented RNA *in situ* hybridization technology developed by ACD, designed for detecting RNA in fixed tissues. We prepared colon fragments by fixing them in 10% NBF for 16 hours, then slicing them into 5-µm sections. Following deparaffinization and rehydration, we adhered to the vendor’s manual for *in situ* hybridization (FISH). The procedure entailed the following steps (1): Pre-treatment with H_2_O_2_, target retrieval, and proteinase plus, among other steps (2). Hybridization of the 16s rRNA probe to the RNA target using a hybridization oven for 2 hours at 40°C (3). Signal amplification through a series of steps, including Amp1, Amp2 and Amp3. Subsequently, we visualized the signal using TSA-fluorescent (TSA-cyanine 3, PerkinElmer LIFE SCIENCES) detection methods. Samples were counterstained with ProLong™ gold Mountant with DAPI (Invitrogen, cat no. p36931) for further analysis. The fluorescent images were captured with a DMC2900 Color Camera using the Leica Thunder Microscope Imager System, manufactured in Wetzlar, Germany. These images were then processed and assembled with Adobe Photoshop. In the final analysis, we focused on attributes like bacterial load, bacterial translocation, and the morphology of the epithelium.

### Bio-plex multiplex immunoassay system

Start by preparing the serum sample matrix according to the assay kit’s guidance and dilute the samples to the specified concentrations. Into the wells of the magnetic bead plate, dispense 50 µL of the diluted samples or standards. Add 50 µL of the premixed bead solution containing cytokine-specific beads to each well, then cover and incubate the plate on a shaker at room temperature for 30 to 60 minutes. After the initial incubation, wash the plate using Bio-Plex Pro™ Wash Buffer. Then, introduce 25 µL of the premixed detection antibody solution to each well and place the plate back on the shaker for another 30 to 60 minutes at room temperature. Following this, wash the plate again using the same wash buffer. Subsequently, add 50 µL of premixed streptavidin-phycoerythrin (SAPE) to each well and let it incubate on the shaker for 10 minutes at room temperature. After this, resuspend the beads with the Bio-Plex Pro™ Assay Buffer and transition the plate to the Bio-Plex Pro™ II or Bio-Plex® 200 system for data acquisition. Employ the Bio-Plex Manager™ software to configure the assay, setting the cytokine standards accordingly. For the final steps, use the Bio-Plex Manager™ software to create standard curves and determine the cytokine concentrations in the samples.

### RNA extraction and quantitative real-time RT-PCR

RNA was extracted from liver tissue using the RNeasy RNA extraction kit from QIAGEN (Hilden, Germany). This RNA was then reverse-transcribed into single-stranded cDNA using the iScript™ cDNA synthesis kit from Bio-Rad Laboratories (Hercules, CA), following the manufacturer’s guidelines. Quantitative real-time PCR was conducted to measure gene transcripts, adhering to a previously described standard protocol (61). The expression levels of specific gene mRNA in samples were calculated using the 2^−ΔΔ^C^T^ method. The 18S rRNA was used as a control gene to calculate ΔCT, where ΔCT = CT-target - CT-18S. The primers for the housekeeping gene *18S rRNA* are as follows: Forward (*18S rRNA*-F) 5′-TGCCC TATCAACTTTCGATG-3′ and Reverse (*18S rRNA*-R) 5′-GATGTGGTAGCCGTT TCTCA-3′. For target genes related to pro-inflammatory cytokines, the primers used include: for *Tnfa*, Forward (*Tnfa*-F) 5′-CCACCACGCTCTTCTGTCTA-3′ and Reverse (*Tnfa*-R) 5′-AGGGTCTGGGCCATAGAACT-3′; and for *Il1B*, Forward (*Il1B*-F) 5′-GAAATGCCACCTTTTGACAGTG-3′ and Reverse (*Il1B*-R) 5′- TGGATGCTCTCATCAGGACAG -3′. The ΔΔCT value represents the CT difference between the normalized sample amount and the normalized calibrator amount.

### ALT and AST activity assays

Aspartate Aminotransferase (AST) and Alanine Aminotransferase (ALT) serve as widely recognized markers for liver injury and systemic inflammation. To determine their levels, we employed the ALT and AST assay kit from Thermo Fisher. Before starting the assay, the spectrophotometer was calibrated to read an absorbance at 340 nm, which corresponds to the maximum absorbance of NADH. The assay was set up in a 96-well plate with each well containing: 20 µl of ALT or AST substrate solution, 20 µl of 2-oxoglutarate solution, and an appropriate volume of ALT or AST enzyme solution, which could be either purified enzyme or a serum sample. After throughly mixing, the plate was incubated at 37°C for 5 minutes. Subsequently, 100 µl of NADH solution and 50 µl of LDH solution were added to each well. After another round of mixing, the plate was placed in the spectrophotometer. Absorbance changes at 340 nm were recorded for 2-3 minutes, reflecting the ΔA/min. The enzyme activity was then calculated using the formula: ALT or AST activity (U/L) = (ΔA/min × Total assay volume × Dilution factor)/(6.22 × Sample volume).

### Seahorse assay

The Seahorse assay is a method for measuring cellular bioenergetics, including mitochondrial respiration and glycolysis, in real-time. To perform the assay, cells were seeded onto a Seahorse assay plate and incubated overnight. On the day of the assay, the cells were washed, and the assay plate was equilibrated to the appropriate temperature and CO2 concentration. Then, a series of compounds (Glucose, Oligomycin, and 2-DG in Glycolytic Stress kit) were added to the plate to sequentially measure basal respiration, glycolysis, glycolytic capacity, and glycolytic reserve. The Seahorse instrument measures the oxygen and proton concentrations in the wells of the assay plate to calculate the cellular respiration and glycolysis rates. Data analysis can be performed using the Seahorse Wave software.

### Flow cytometry analysis

Splenocytes were harvested and red blood cells were eliminated using the RBC lysis buffer from BD. To block Fc receptors and reduce non-specific antibody binding, the isolated splenocytes were treated with FcBlock (BD Biosciences) for 15 minutes at 4°C. Subsequently, cells were stained with a mixture of fluorochrome-conjugated antibodies: FITC-conjugated CD45 at a 1:100 dilution and APC-conjugated Annexin V at a 1:200 dilution (both from Bio-Rad). The stained cells were then analyzed on a BD LSRFortessa flow cytometer, utilizing BD FACSDiva software for data acquisition (BD Biosciences). Compensation adjustments and data analyses were carried out post-acquisition using FlowJo software (TreeStar, Ashland, OR). To ensure the specificity of staining and accurate gating, “Fluorescence minus one” controls were employed when required. Identification of specific cell populations was done through a sequential gating strategy, which is further elaborated upon in the Result section. Expression levels of activation markers are denoted as median fluorescence intensity (MFI).

### Human IBD patient database bioinformatic analysis

Microarray data of human IBD patients (Databases GSE59071, GSE75214, and GSE92415), accessible from databases GEO, undergo pre-processing and normalization (e.g., RMA or Quantile normalization) to ensure quality and consistency. Biomarkers drawn from various panels of the human sepsis Bio-plex detection assay (including IL1B, LCN2, CXCL8, IL6, IL1A, ICM1, CCL3, CCL4, THBS1, GZMB, FAS, VCAM1, MMP8, TNF, LIF, HSPA4, ELANE, RETN, and FASLG) were analyzed using human IBD databases. Differentially expressed genes (DEGs) between IBD and healthy control colonic samples are determined using statistical tests, with adjustments for multiple testing. Further, changes in gene expression within the colon were calculated and log2 fold-change (FC) values were used to generate a heatmap. Red signifies increased expression in inflamed samples compared to controls, while green indicates decreased expression. In another set analysis of human IBD blood samples, significant changes in blood gene expression were depicted in a volcano plot drawn from the GSE92415 database, the pink dot indicates the adjust *p* < 0.05, and the red dot reveals adjust *p* < 0.01. Throughout, the analysis aligns with existing biological insights into IBD, correlating identified genes and pathways with known mechanisms and exploring potential therapeutic implications.

### Statistical analysis

In this study, animal experiments were conducted by repeating each experiment two or three times, with each repetition involving groups of either four or six mice. For *in vitro* experiments, the term ‘biological replicates’ refers to the number of individual mice used, and ‘technical replicates’ denotes the repeated measurements or samples taken from a single mouse. Data were analyzed using GraphPad Prism 6 software and are presented as mean ± standard error of the mean (s.e.m.). To check for normality, datasets were visually inspected using Q-Q plots. Statistical comparisons between two groups were conducted using the two-tailed Student’s t-test, appropriate for parametric data. For comparisons involving multiple groups, a one-way analysis of variance (ANOVA) was applied. The Pearson correlation coefficient was used to determine the correlation between variables. Additionally, Kaplan–Meier survival analysis was performed, with the log-rank test employed to compare survival rates across different groups. A p-value of less than 0.05 (two-sided) was considered indicative of a statistically significant difference.

## Data availability statement

The original contributions presented in the study are included in the article/supplementary materials, further inquiries can be directed to the corresponding author/s.

## Ethics statement

Ethical approval was not required for the study involving humans in accordance with the local legislation and institutional requirements. Written informed consent to participate in this study was not required from the participants or the participants’ legal guardians/next of kin in accordance with the national legislation and the institutional requirements. The animal study was approved by Animal Care Center (ACC) at the University of Illinois at Chicago. The study was conducted in accordance with the local legislation and institutional requirements.

## Author contributions

XW: Conceptualization, Formal analysis, Funding acquisition, Investigation, Methodology, Supervision, Validation, Writing – original draft, Writing – review & editing. CD: Investigation, Writing – review & editing. SS: Investigation, Writing – review & editing. LT: Investigation, Writing – review & editing. HG: Investigation, Writing – review & editing. H-FB: Investigation, Writing – review & editing. X-DT: Conceptualization, Funding acquisition, Supervision, Writing – review & editing.
